# 4185 Assessing Barriers to Retention in Care Continuum Among HCV Positive Homeless Adults of New Orleans

**DOI:** 10.1017/cts.2020.369

**Published:** 2020-07-29

**Authors:** Riley Eli Santiago, Janna Wisniewski

**Affiliations:** 1 Tulane University School of Medicine- LA CaTS; 2 Tulane University

## Abstract

OBJECTIVES/GOALS: This study has two primary aims: 1) evaluate points of success and failure in connecting hepatitis C virus (HCV) positive homeless patients to care following a preliminary positive rapid HCV test result, and 2) describe the barriers cited by patients who drop out at each step in the care continuum. METHODS/STUDY POPULATION: A retrospective longitudinal analysis of adult (18 years or older) homeless individuals accessing shelter at six homeless shelters in New Orleans, LA was conducted. Every patient who came through a testing site received a survey collecting information on demographics, barriers to healthcare, and recent utilization of health services. A retrospective chart review of hospital and homeless clinic medical records was used to track patient linkage to care and their progress through the HCV care continuum. We defined successful linkage to care as attendance at the first scheduled follow-up appointment for treatment with a primary care physician. RESULTS/ANTICIPATED RESULTS: A total of 1719 unique patients were identified from August 2016 through August 2019 which included 36% self-identified as African American/Black, 55% identified as White and 8% identified as mixed-race or other. A total of 24% of individuals reported no insurance coverage while 66% of patients reported having insurance. Overall, 85 patients reported they experienced no barriers to healthcare. Of those who reported barriers, 44% reported trouble with finances or insurance, 22% transportation, 18% personal drug use, 9% personal alcohol use, and 7% reported a distrust of healthcare providers or the system. Other barriers included long wait times, distance, and recent incarceration. DISCUSSION/SIGNIFICANCE OF IMPACT: Although screening for HCV is readily available, barriers exist which prevent diagnosis and treatment. We implemented a HCV testing and linkage-to-care program between local homeless shelters and health centers in New Orleans in an effort to reduce HCV-related morbidity and mortality.

